# Supplementary material for: Application of Digital Holographic Microscopy to Analyze Changes in T-Cell Morphology in Response to Bacterial Challenge
Source: Cells. 2023 Feb 27;12(5):762. doi: 10.3390/cells12050762 (PMC10000559; doi:10.3390/cells12050762)
Supplement: Supplementary file 1 [file cells-12-00762-s001.zip › cells-2209635-supplementary.pdf]

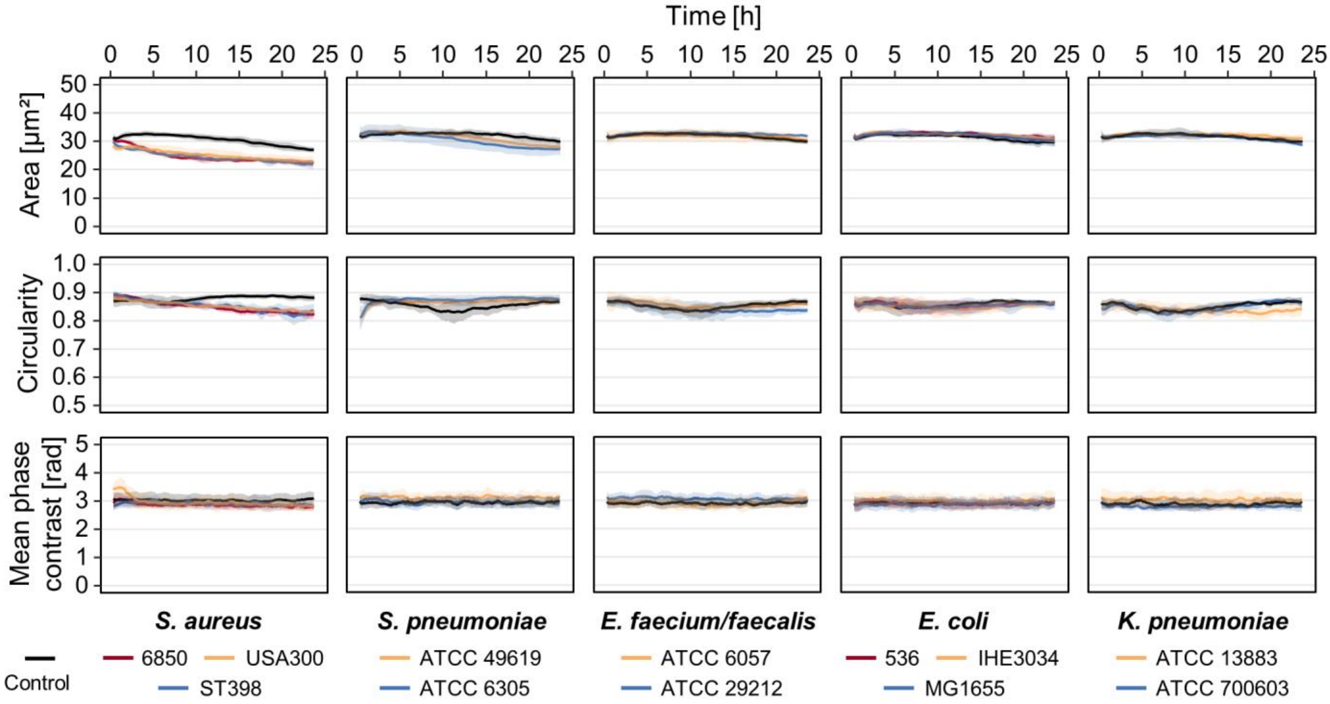

Figure S1. Morphological changes of T-cells treated with 5 % culture supernatant. Primary T-cells were treated with diluted culture supernatants (5 % v/v) derived from different bacteria or left untreated as control (black lines). Time-lapse DHM was applied and the resulting phase contrast images were analyzed for single cell area, circularity and mean phase contrast per cell. Results represent mean (solid lines) ± SD (shading) from at least three independent experiments. The curves were smoothed using moving averages with a window size of 15 measuring points.

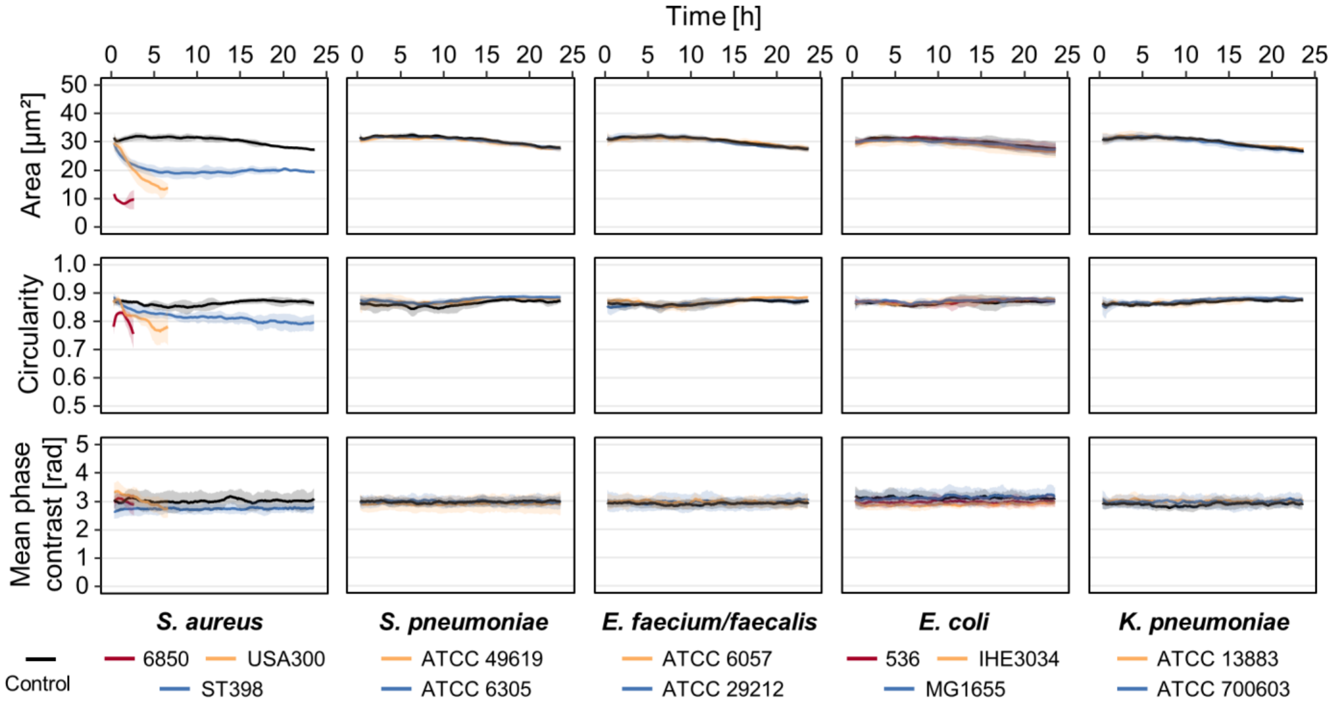

Figure S2. Morphological changes of T-cells treated with bacterial MVs. Primary T-cells were exposed to bacterial MVs (2×10^9 particle/mL) derived from various bacterial species and strains. Untreated cells served as control (black lines). Cellular morphological changes were captured using time-lapse DHM and single cell area, circularity and mean phase contrast per cell were analyzed. The results are presented as mean (solid lines) ± SD (shading) from at least three biological replicates. The curves were smoothed using moving averages with a window size of 15 measuring points.
